# Synthesis, Characterization, Antimicrobial Activity, and Genotoxicity Assessment of Two Heterocyclic Compounds Containing 1,2,3-Selena- or 1,2,3-Thiadiazole Rings

**DOI:** 10.3390/molecules24224082

**Published:** 2019-11-12

**Authors:** Mousa L. Al-Smadi, Reem Mansour, Amjad Mahasneh, Omar F. Khabour, Majed M. Masadeh, Karem H. Alzoubi

**Affiliations:** 1 Department of Applied Chemical Science, Faculty of Science and Arts, Jordan University of Science and Technology, Irbid 22110, Jordan; reem@aci.com.jo; 2 Department of Biotechnology and Genetic Engineering, Jordan University of Science and Technology, Irbid 22110, Jordan; amjada@just.edu.jo; 3 Department of Medical Laboratory Sciences, Faculty of Applied Medical Sciences, Jordan University of Science and Technology, Irbid 22110, Jordan; Khabour@just.edu.jo; 4 Department of Pharmaceutical Technology, Jordan University of Science and Technology, Irbid 22110, Jordan; mmmasadeh@just.edu.jo; 5 Department of Clinical Pharmacy, Jordan University of Science and Technology, Irbid 22110, Jordan; khalzoubi@just.edu.jo

**Keywords:** thiadiazole rings, heterocyclic compounds, synthesis, characterization, antimicrobial activity, genotoxicity

## Abstract

New 1,2,3-thiadiazole and 1,2,3-selenadiazole derivatives, (4-[4-((4-bromobenzyl)oxy)-phenyl]-1,2,3-thiadiazole (**5a**), 4-[4-((4-chlorobenzyl)oxy)-phenyl]-1,2,3-thiadiazole (**5b**)), (4-[4-((4-bromobenzyl)oxy)-phenyl]-1,2,3-selenadiazole (**6a**), and 4-[4-((4-chlorobenzyl)oxy)-phenyl]-1,2,3-selenadiazole (6b)), were prepared and screened in vitro for their antimicrobial activity against various pathogenic microbes. In addition, two compounds (**5a** and **6a**) were examined for their in vivo genotoxicity using rats and an 8-hydroxy-2′-deoxyguanosine (8-OHdG) assay. Compounds **5a** and **5b** were found to be highly active against Gram-positive and Gram-negative bacteria. In addition, a significant inhibition of urinary 8-OHdG level (50.2%) was observed upon treatment of animals with 500 mg/kg body weight (b.w.) of compound **6a** (*p* < 0.0001). However, compound **5a** increased urinary 8-OHdG levels. The lethal dose (LD_50_) values for compounds **5a** and **6a** were determined by an up-and-down procedure (OECD 425; OECD 1998), which showed that these compounds are safe, since the LD_50_ was >5000 mg/kg b.w. Thus, the tested compounds might have the potential for use as antibiotics, since they have low genotoxicity and strong antimicrobial activity.

## 1. Introduction

Many substituted 1,2,3-thiadiazoles and derivatives were prepared, but few of them showed high antibacterial activity [1]. The concept of isosteric exchange was long used as a tool for modifying the activity of biologically important molecules. One such important isosteric pair is sulfur and selenium, which were incorporated as heteroatoms in the heterocyclic system [2]. The 1,2,3-thiadaizole and 1,2,3-selenadiazole compounds were reported to possess antibacterial, antifungal [1,3,4], antiviral [5], and anticancer [6] activities. The current study assessed the activity of newly prepared 1,2,3-thiadiazole (**5a**, **5b**) and 1,2,3-selenadiazole (**6a**, **6b**) derivatives against representative common pathogenic bacteria and fungi. In addition, the genotoxicity of the newly synthesized compounds was measured using the 8-hydroxy-2′-deoxyguanosine (8-OHdG) assay in rat’s urine. The 8-OHdG assay is one of the most reliable and abundant biomarkers of DNA damage because it reflects extremely low levels of oxidative damage [7]. In addition, the assay can be used for both in vivo and in vitro studies [8,9], as well as in many diseases, including bladder and prostate cancer, cystic fibrosis, atopic dermatitis, and rheumatoid arthritis [7,10,11,12]. The results of this study are expected to strengthen current knowledge regarding the biological activities of 1,2,3-thiadiazole and 1,2,3-selenadiazole compounds, to explore the potential use of these compounds as antibiotics, and to establish structure–activity relationships for such use.

## 2. Result and Discussion

**Scheme 1:**
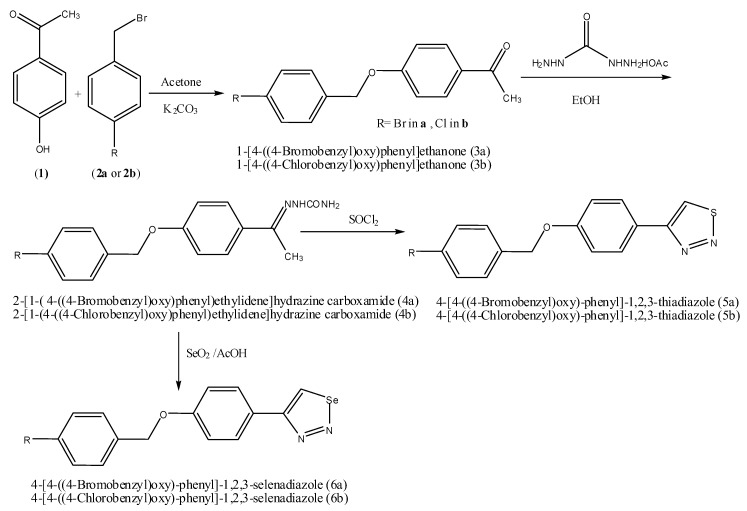
Synthesis of compounds **3a**–**6a** and **3b**–**6b**.

New 1,2,3-thiadiazole, and 1,2,3-selenadiazole derivatives were prepared using the methods that were previously established by Hurd and Mori for 1,2,3-thiadiazole preparation and by Lalezari et al. for 1,2,3-selenadizole preparation [13,14]. The mechanisms of synthesis of both 1,2,3-thiadiazole and 1,2,3-selenadiazole were previously reported. In both methods, α-methyl ketone derivatives (**3a**, **3b**) are formed by an S_N_2-nucleophilic substitution reaction of 4-hyroxyacetophenone and 4-halobenzyl bromide in basic medium. The semicarbazone derivatives (**4a**, **4b**) which were prepared from the condensation reaction of the corresponding ketones (**3a**, **3b**) and semicarbazide hydrochloride were used in the preparation of the 1,2,3-thiadiazole (**5a**, **5b**) and 1,2,3-selenadiazole derivatives (**6a**, **6b**). The total synthesis of the ketones, semicarbazones, 1,2,3-thiadiazole derivatives, and 1,2,3-selenadiazole derivatives is illustrated in Scheme 1. The percentage yields of all steps were optimized, and the overall yields for the reaction sequences **2a** → **3a**, **2b** → **3b**, **3a** → **4a**, **3b** → **4b**, **4a** → **5a**, **4b** → **5b**, **4a** → **6a**, and **4b** → **6b** are given in Table 1. The activity of the heterocyclic compounds **5a**, **5b**, **6a**, and **6b** was tested against human pathogenic microbes including Gram-positive *Staphylococcus aureus*, Gram-negative *Escherichia coli*, local resistant *Pseudomonas aeruginosa* and *Pseudomonas aeruginosa* (ATCC 27853), and *Candida albicans* by the hole diffusion method. As shown in Table 2, two of the tested heterocyclic compounds (**5a** and **5b**) were active at 0.01 gm/mL concentration. The minimum active concentrations of the compounds that gave positive results (**5a** and **5b**) in the hole method were determined (Table 3). The results showed that the sensitivity of *Staphylococcus aureus* and *Candida albicans* to the heterocyclic compounds **5a** and **5b** was high, with a 5–10-mm-diameter inhibition zone at 5 mg/mL concentration, as indicated in Table 3. The solvent showed no activity against any of the tested pathogens. As indicated from the presented data, the synthetic heterocyclic compounds **5a** and **5b** show high potential as novel antimicrobial agents. In addition, these heterocyclic compounds were active against *Staphylococcus aureus* (Gram-positive bacterium) and *Candida albicans* (fungus).

The two compounds (**5a** and **6a**) that showed the best antimicrobial activity were selected for standard toxicity testing. The two compounds differed only in the type of heteroatom present in the ring (S or Se); thus, their results could help to determine the effect of different heteroatoms on the toxicity of the whole compound. The lethal dose (LD_50_) was determined by injecting dosages ranging from 0 mg/kg to 5000 mg/kg of compounds **5a** and **6a** to rats. No clinical change or toxicity effect was observed in the rats up to 5000 mg/kg dosage. Thus, both compounds were considered safe and of low toxicity (Table 4 and Table 5) [15].

**Figure 1:**
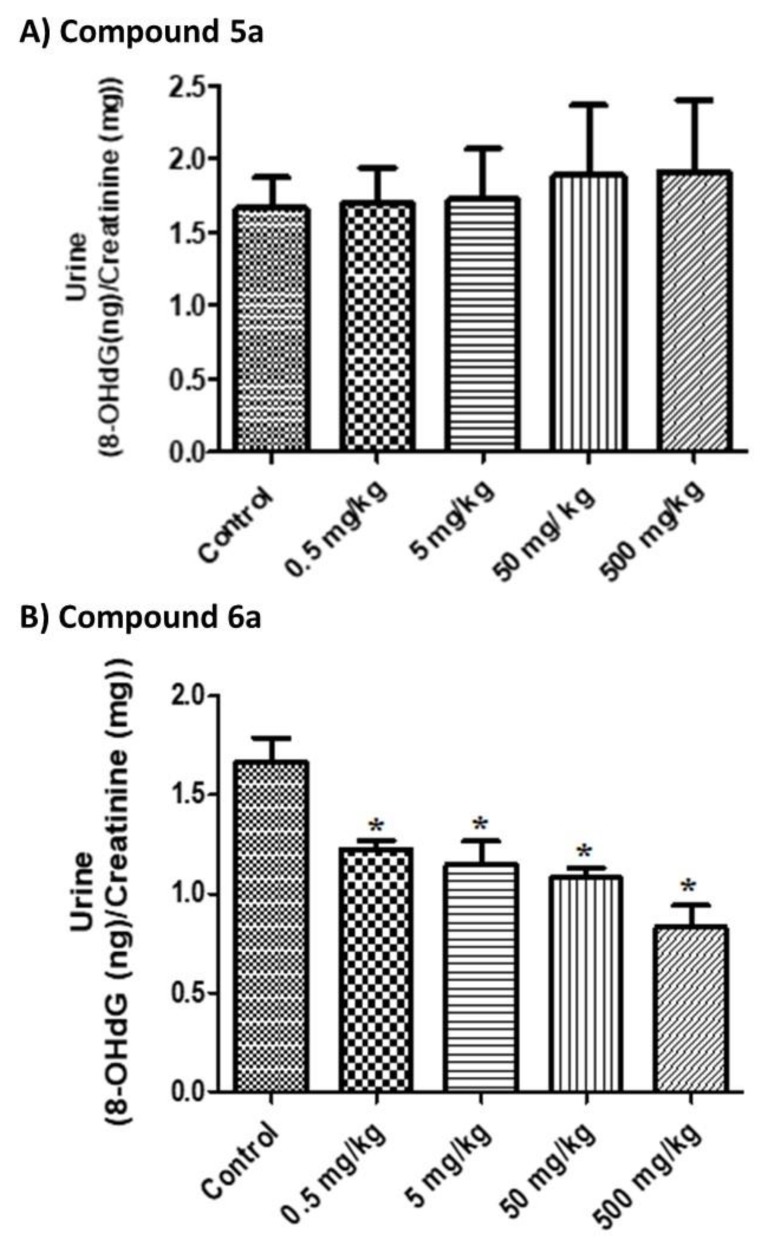
The genotoxicity of the (**A**) 1,2,3-thiadiazole (**5a**) and (**B**) selenadiazole (**6a**) compounds; * significant difference from control at *p* < 0.05 using one-way ANOVA and Tukey’s post hoc test.

The 8-OHdG compound is a deoxyguanosine oxidation product of DNA. Extensive studies over the past three decades established its biological significance. Since 8-OHdG is produced in cellular DNA, it represents an indicator of oxidative stress and genotoxicity of chemical agents both in vivo and in vitro [16]. Environmental pollutants, such as polyaromatic hydrocarbons, metal fumes, and fly ash, increase the level of urinary 8-OHdG. Oxygen radical-related diseases like cancer, and cardiovascular and periodontal conditions have high urinary 8-OHdG levels [17,18]. The 8-OHdG levels in urine and lymphocyte DNA damage are also well correlated, and the 8-OHdG/creatinine values of 24-h urine samples and overnight urine (early morning urine) show a good correlation [19]. Therefore, we tested the impact of two (**5a** and **6a**) of the synthesized compounds on the 8-OHdG level using an in vivo animal model. We examined only two of the synthesized compounds due to budget limitations, as such experiments are costly. The results are shown in Figure 1. The results showed that compound **5a** is not genotoxic, as no significant increases in the urinary 8-OHdG level were observed after treatment (*p* > 0.05). On the other hand, compound **6a** significantly reduced the basal level of 8-OHdG (*p =* 0.0001) by about 50.18%, which indicates a potential effect of this compound with regard to in vivo oxidative DNA damage. Thus, the selenium derivative (compound **6a**) is a more potent antioxidant than the sulfur derivative (compound **5a**), whereas the structures of the two compounds are similar. It is worth mentioning that, in the genotoxicity experiments, no positive control was used due to budget limitations. Therefore, the current genotoxicity findings should be confirmed in a more controlled experimental design.

The finding that compound **6a** showed a strong protective effect with regard to in vivo oxidative DNA damage is interesting and points to the potential use of this compound as an agent to overcome oxidative stress associated with cellular metabolism and disease conditions. The mechanism via which compound **6a** protected the animals against oxidative damage requires further investigation. The body develops different protective mechanisms against oxidative damage. These include various antioxidant enzymes such as superoxide dismutase (SOD), catalase, and glutathione peroxidase, as well as antioxidant vitamins (C and E) that can directly scavenge reactive oxygen species (ROS). Glutathione peroxidase (GPx) is considered an important antioxidant enzyme, and it contains a selenium atom in its active domain. GPx effectively scavenges and eliminates H_2_O_2_ both in vitro and in vivo. In addition, previous studies showed that selenium compounds such as selenoproteins protect cells against oxidative stress; accordingly, a variety of selenium compounds may effectively scavenge and eliminate ROS [20]. On the other hand, certain compounds like thioureas that impact the twitch tension of the rat diaphragm upon stimulation of the phrenic nerve diaphragm also scavenge O_2_^−^. Thus, since compound **6a** has a selenium atom in its heterocyclic ring, it may act as a scavenger of radicals to prevent oxidative DNA damage. Alternatively, compound **6a** may work by enhancing the activity of antioxidative enzymes.

Notably, in vivo injected compounds may be subjected to metabolism through specialized enzymatic systems in the body that often convert lipophilic chemical compounds into more readily excreted polar products [21,22]. Metabolism of a compound can result in an increase or a decrease in its toxicity [23]. Therefore, it is possible that compounds **5a** and **6a** entered different metabolic pathways in the body that differentially modulated their structure and/or toxicity. Therefore, the inhibition of urinary 8-OHdG by compound **6a** may have resulted through releasing the selenium atom under metabolic conditions in rats. However, the exact mechanism via which compound **6a** protects against oxidative damage will be the matter of future studies.

## 3. Materials and Methods

### 3.1. General

The solvents were purified by standard procedures. The melting points (m.p.) were determined using an electrothermal digital melting point apparatus and were uncorrected. Infrared (IR) spectra were recorded using an IR Prestige-21 Fourier-transform spectrophotometer version 1.50 (ν_max_ in cm^−1^). Proton nuclear magnetic resonance (^1^H-NMR) spectra were recorded at 400 MHz (JEOL, JNM-ECP400, FT-NMR system; 200 MHz with AC200 instrument from Bruker company). Tetramethylsilane (TMS) was used as an internal reference. Carbon nuclear magnetic resonance (^13^C-NMR) spectra were recorded at 100 MHz (JEOL, JNM-ECP400, FT-NMR system; 50 MHz with AC200 Instrument from Bruker company). Ultraviolet (UV) spectra were recorded using a UV-1700 Pharma. Spec. Shimadzu corporation spectrometer; the wavelength was recorded in nanometer (nm) units with absorption (Abs). The mass spectra were carried out using instruments MAT CH7A from Varian (electron ionization (EI): 70 eV ionization energy) and MAT95 from Finnigan (field desorption (FD): 5 kV ionizing energy). The signals were given as *m*/*z* with the relative intensity between brackets. Analytical thin-layer chromatography (TLC) was carried out using TLC silica plates (0.2 mm) from Merck Company. The elemental analysis was carried out using an elemental analyzer from NCHS Euro 3000, Italy. Furthermore, 4-hydroxyacetophenone, selenium dioxide, and thionyl chloride were obtained from ACROS. These chemicals were used without further purification. Semicarbazide hydrochloride was obtained from Schuchardt (Merck). Sodium acetate anhydrous (extra pure), potassium carbonate, and sodium bicarbonate were obtained from Scharalau, while 4-chloro benzyl bromide **(2b)** and 4-bromo benzyl bromide (**2a**) were obtained from Aldrich. The animals used in the study were six-month-old Fischer male rats (200–250 g). The animals were reared in the animal care facility at Jordan University of Science and Technology (JUST) at 24 ± 1 °C with a 12-h/12-h light/dark cycle (light onset at 7:00 a.m.). The study procedure was approved by the animal care and use committee at JUST. Statistical analysis was performed using Graph Pad Prism software (version 4.0; Graph Pad Software, Inc., La Jolla, CA, USA). Multigroup analysis was conducted with analysis of variance (ANOVA), and significant group effects were further analyzed with Dunnett’s multiple comparison test. Significant differences were examined at a *p*-value of 0.05. Figures S1–S20 (Supplementary Materials) show the NMR and IR spectra of various synthesized compounds.

### 3.2. General Procedure for the Preparation of Ketones ***3a**–**3b***

A mixture of 4-hyroxyacetophenone (one equivalent), 4-bromobenzyl bromide (**2a**), or 4-chlorobenzyl bromide (**2b**) (one equivalent), potassium carbonate (one equivalent), and a few drops of Aliquat 336 in 100 mL of dry acetone was refluxed for 60 h. The reaction was followed by TLC in chloroform until completion. The reaction mixture was cooled, the precipitated salt was removed by simple filtration, and the solvent was evaporated in vacuum to give a pale-yellow oil, which crystallized quickly at room temperature. The solid was washed using a small amount of cold ethanol and dried under vacuum.

### 3.3. 1-[4-((4-Bromobenzyl)oxy) phenyl]ethanone *(**3a**)*

White solid, yield 98%, m.p. (°C): 114–116; IR (KBr) (cm^−1^): C–H group at 2940, 2900, 2876; C=O at 1675; C=C at 1600, 1580; C–O at 1256. UV spectra (chloroform) λ _max_: 270 (nm). ^1^H-NMR (acetone-*d*_6_) (ppm), 2.51(3H, s, COCH_3_), 7.97 (2H, d, *J*_AB_ = 8.32 Hz, H_A_, –C_6_H_4_–COCH_3_), 7.09 (2H, d, *J*_AB_ = 8.32 Hz, H_B_, –C_6_H_4_–COCH_3_), 5.22 (2H, s, BrArCH_2_O), 7.45 (2H, d, *J*_AB_ = 8.8 Hz, H_A_, Br–C_6_H_4_–CH_2_O–), 7.58 (2H, d, *J*_AB_ = 8.8 Hz, H_B_, Br–C_6_H_4_–CH_2_O–). ^13^C-NMR (acetone-*d*_6_) (ppm) 26.41 (COCH_3_), 196.39 (COCH_3_), 130.4, 131.6, 115.4, 163.2 (–C_6_H_4_–COCH_3_), 69.86 (BrArCH_2_O), 137.2, 131.2, 132.4, 122.0 (Br–C_6_H_4_–CH_2_O–). FDMS *m*/*z*: 305.1 [M^+^, 100]. Analyzed percentage calculated for BrC_15_H_13_O_2_: C 59.04, H 4.29; found C 58.85, H 4.15.

### 3.4. 1-[4-((4-Chlorobenzyl)oxy)phenyl]ethanone *(**3b**)*

White solid, yield: 97%, m.p. (°C): 99–102; IR (KBr) (cm^−1^): C–H group at 2896, 2840; C=O at 1666; C=C at 1602; C-O at 1252. UV spectra (chloroform) λ _max_: 269 (nm). ^1^H-NMR (acetone-*d*_6_) (ppm): 2.45 (3H, s, COCH_3_), 7.93 (2H, d, J_AB_ = 8.3 Hz, H_A_, –C_6_H_4_–COCH_3_), 7.07 (2H, d, J_AB_ = 8.32 Hz, H_B,_ –C_6_H_4_–COCH_3_), 5.21 (2H, s, ClArCH_2_O–), 7.41 (2H, d, J_AB_ = 8.7 Hz, H_A_, Cl–C_6_H_4_–CH_2_O–), 7.49 (2H, d, J_AB_ = 8.7 Hz, H_B_, Cl–C_6_H_4_–CH_2_O–). ^13^C-NMR (acetone-*d*_6_) (ppm): 25.58 (COCH_3_), 195.54 (COCH_3_), 129.3, 131.0, 114.5, 162.4 (–C_6_H_4_–COCH_3_), 69.0 (ClArCH_2_O), 135.9, 130.4, 133.03, 128.6 (Cl–C_6_H_4_–CH_2_O–). FDMS *m/z*: 260.2 [M^+^, 100]. Analyzed percentage calculated for ClC_15_H_13_O_2_: C 69.10, H 5.03; found C 68.82, H 4.83.

### 3.5. General Procedure for the Preparation of Semicarbazones *(**4a**–**4b**)*

A mixture of semicarbazide hydrochloride (one equivalent) and sodium acetate (one equivalent) was dissolved in 50 mL of absolute ethanol and heated to reflux for 30 min. The mixture was filtered while hot to remove precipitated sodium chloride salt. An equivalent amount of ketone (**3a** or **3b**) was added to the hot filtrate solution. This mixture was refluxed for 1 h and water generated was continuously removed using a succulent with MgSO_4_ as a drying agent. After 30 min, the reaction started producing a white precipitate that increased with time. After reaction completion, the reaction mixture was cooled to room temperature; then, the precipitated semicarbazone was filtered off and washed with cold ethanol and dried under vacuum.

### 3.6. 2-[1-(4-((4-Bromobenzyl)oxy) phenyl) ethylidene]hydrazine Carboxamide *(**4a**)*

White solid, 85% yield, m.p. (°C): 220–222; IR (KBr) (cm^−1^): NH_2_ at 3340, 3300; NH at 3500; C–H at 3200, 2970, 2958, 2890; C=O at 1689; C=C, C=N at 1610, 1580, 1720; C-O at 1258. UV (chloroform) λ _max_: 281.5 (nm). ^1^H-NMR (dimethyl sulfoxide (DMSO)-*d*_6_) ppm: 5.53 (2H, s, NHCONH_2_), 8.29 (1H, s, –C(CH_3_)(NNHCONH_2_)), 1.23 (3H, s, –C(CH_3_)(NNHCONH_2_)), 6.85 (2H, d, *J*_AB_ = 8.2 Hz, H_A_, –C_6_H_4_– (C)(CH_3_)(NNH(CO)NH_2_)), 6.05 (2H, d, *J*_AB_ = 8.2 Hz, H_B_, –C_6_H_4_– (C)(CH_3_)(NNH(CO)NH_2_)), 4.21 (2H, s, BrArCH_2_O–), 6.49 (2H, d, J_AB_ = 8.7 Hz, H_A_, Br–C_6_H_4_–CH_2_O–), 6.67 (2H, d, *J*_AB_ = 8.7 Hz, H_B_, Br–C_6_H_4_–CH_2_O–). ^13^C-NMR (DMSO-*d*_6_) ppm: 157.31 (NHCONH_2_), 30.63 (–C(CH_3_)(NNHCONH_2_)), 143.7 (–C(CH_3_)(NNHCONH_2_)), 131.11, 120.87, 114.38, 158.37 (–C_6_H_4_–(C)(CH_3_)(NNH(CO)NH_2_)), 68.33 (BrArCH_2_O), 136.44, 129.72, 127.32, 131.3 (Br–C_6_H_4_–CH_2_O–). FDMS *m*/*z*: 361.1 [M^+^, 100]. Analyzed percentage calculated for BrC_16_H_17_O_2_N_3_: C 53.05, H 4.42, N 11.60; found C 52.89, H 4.35, N 11.50.

### 3.7. 2-[1-(4-((4-Chlorobenzyl)oxy)phenyl)ethylidene]hydrazine Carboxamide *(**4b**)*

White solid, 82% yield, m.p. (°C): 203–205; IR (KBr) (cm^−1^): NH_2_ at 3380, 3290; NH at 3500; C–H at 3150, 2970, 2940, 2868; C=O at 1680; C=C, C=N at 1610, 1586, 1750; C–O at 1252. UV (chloroform) λ _max_: 282.5 (nm). ^1^H-NMR (DMSO-*d*_6_) ppm: 5.52 (2H, s, NHCONH_2_), 8.28 (1H, s, –C(CH_3_)(NNHCONH_2_)), 1.23 (3H, s, –C(CH_3_)(NNHCONH_2_)), 6.86 (2H, d, *J*_AB_ = 8.6 Hz, H_A_, –C_6_H_4_– (C)(CH_3_)(NNH(CO)NH_2_)), 6.06 (2H, d, *J*_AB_ = 8.6 Hz, H_B_, –C_6_H_4_–(C)(CH_3_)(NNH(CO)NH_2_)), 4.23 (2H, s, ClArCH_2_O–), 6.53 (2H, d, *J*_AB_ = 8.7 Hz, H_A_, Cl–C_6_H_4_–CH_2_O–), 6.56 (2H, d, *J*_AB_ = 8.7 Hz, H_B_, Cl–C_6_H_4_–CH_2_O–). ^13^C-NMR (DMSO-*d*_6_) ppm: 157.3 (NHCONH_2_), 30.65 (–C(CH_3_)(NNHCONH_2_)), 143.73 (–C(CH_3_)(NNHCONH_2_)), 131.1, 127.3, 114.39, 158.4 (–C_6_H_4_– (C)(CH_3_)(NNH(CO)NH_2_)), 68.3 (ClArCH_2_O), 136.03, 129.43, 128.4, 132.35 (Cl–C_6_H_4_–CH_2_O–). FDMS *m*/*z*: 318.1 [M^+^, 100]. Analyzed percentage calculated for ClC_16_H_17_O_2_N_3_: C 60.28, H 5.33, N 13.12; found C 60.34, H 4.97, N 13.07.

### 3.8. General Procedure for the Preparation of 1,2,3-Thiadiazoles *(**5a**–**5b**)*

Compounds **4a** or **4b** (11 mmol) were slowly added to thionyl chloride (16 mmol) in several portions with vigorous stirring. Then, the mixture was stirred overnight at room temperature until no more hydrogen chloride was produced. The excess thionyl chloride was removed under vacuum. The remaining residue was washed with several portions of petroleum ether (60–70) and then dried under vacuum.

### 3.9. 4-[4-((4-Bromobenzyl)oxy)-phenyl]-1,2,3-thiadiazole *(**5a**)*

Yellow solid, 98% yield, m.p. (°C): 178 (decomposed); IR (KBr) (cm^−1^): C–H at 2950, 3100; C=C at 1611, 1599; C=C–N at 1724; N=N at 1470, 1406; C–O at 1252. UV (chloroform) λ_max_: 260 (nm), 309 (nm). ^1^H-NMR (chloroform-*d*_1_) ppm: 8.52 (1H, s, 1,2,3-thiadiazole), 7.97 (2H, d, *J*_AB_ = 8.04 Hz, H_A,_ –C_6_H_4_)–(C_2_N_2_S)), 7.06 (2H, d, *J*_AB_ = 8.04 Hz, H_B,_ –C_6_H_4_)–(C_2_N_2_S)), 5.08 (2H, s, BrArCH_2_O), 7.32 (2H, d, *J*_AB_ = 8.44 Hz, H_A,_ Br–C_6_H_4_–CH_2_O–), 7.51 (2H, d, *J*_AB_ = 8.44 Hz, H_B,_ Br–C_6_H_4_–CH_2_O–). ^13^C-NMR (chloroform-*d*_1_) ppm: 162.83 (C_4_), 129.33 (C_5_) (1,2,3-thiadiazole), 124.3, 128.82, 115.70, 159.65 (–C_6_H_4_)–(C_2_HN_2_S), 69.59, BrArCH_2_O, 135.88, 129.07, 132.05, 122.30 (Br–C_6_H_4_–CH_2_O–). FDMS *m*/*z*: 347.2 [M^+^, 100]. Analyzed percentage calculated for BrC_15_H_11_O N_2_ S: C 51.89, H 3.19; found C 51.79, H 3.08.

### 3.10. 4-[4-((4-Chlorobenzyl)oxy)-phenyl]-1,2,3-thiadiazole *(**5b**)*

Yellow solid, 97% yield, m.p. (°C): 152 (decomposed); IR (KBr) (cm^−1^): C–H at 3070, 2904, 2844; C=C at 1600, 1580; C=C–N at 1722; N=N at 1460, 1408; C–O at 1245. UV (chloroform) λ _max_: 259, 309 (nm). ^1^H-NMR (chloroform-*d*_1_) ppm: 8.52 (1H, s, 1,2,3-thiadiazole), 7.13 (2H, d, *J*_AB_ = 8.4 Hz, H_A,_ –C_6_H_4_)–(C_2_N_2_S)), 6.27 (2H, d, *J*_AB_ = 8.4 Hz, H_B,_ –C_6_H_4_)–(C_2_N_2_S)), 5.10 (2H, s, ClArCH_2_O), 6.53 (2H, d, *J*_AB_ = 8.7 Hz, H_A,_ Cl–C_6_H_4_–CH_2_O–), 6.58 (2H, d, *J*_AB_ = 8.7 Hz, H_B,_ Cl–C_6_H_4_–CH_2_O–). ^13^C-NMR (chloroform-*d*_1_) ppm: (C_4_), 162.73, (C_5_), 129.0 (1,2,3-thiadiazole), 128.71, 128.95, 115.6, 159.57, (–C_6_H_4_)–(C_2_HN_2_S), 69.59, ClArCH_2_O, 135.88, 128.97, 134.1, 124.19 (Cl–C_6_H_4_–CH_2_O–). FDMS *m/z*: 302.9 [M^+.^, 100]. Analyzed percentage calculated for ClC_15_H_11_O N_2_ S: C 59.50, H 3.64; found C 59.01, H 3.55.

### 3.11. General Procedure for the Preparation of 1,2,3-Selenadiazoles *(**6a**–**6b**)*

Semicarbazone (**4a** or **4b**) (one equivalent) was dissolved in 50 mL of glacial acetic acid with vigorous stirring and gentle heating at 35–40 °C. The solution was treated with selenium dioxide powder (1.35 equivalents). After 2 min, the mixture color became red. The reaction mixture was kept under gentle heating with vigorous stirring overnight. TLC showed that the reaction was complete. The precipitated solid was removed by simple filtration. The filtrate was poured over ice water and extracted with chloroform (50 mL × 3). The combined organic layer was washed with saturated sodium hydrogen carbonate solution and then dried using magnesium sulfate. The solvent was removed by vacuum, and the remaining product was recrystallized from acetone.

### 3.12. 4-[4-((4-Bromobenzyl)oxy)-phenyl]-1,2,3-selenadiazole *(**6a**)*

Off-white to light-brown solid, 60% yield, m.p. (°C): 159 (decomposed); IR (KBr) (cm^−1^): C–H at 3080, 2900, 2870; C=C at 1610, 1582; N=N at 1480, 1460. UV (chloroform) λ_max_: 255, 332.5 (nm). ^1^H-NMR (chloroform-*d*_1_) ppm: 9.25 (1H, s, 1,2,3-selenadiazole), 7.97 (2H, d, J_AB_ = 8.2 Hz, H_A,_ –C_6_H_4_)–(C_2_N_2_Se)), 7.06 (2H, d, *J*_AB_ = 8.2 Hz, H_B,_ –C_6_H_4_)–(C_2_N_2_Se)), 5.08 (2H, s, BrArCH_2_O–), 7.31 (2H, d, *J*_AB_ = 8.7 Hz, H_A,_ Br–C_6_H_4_–CH_2_O–), 7.51 (2H, d, *J*_AB_ = 8.7 Hz, H_B,_ Br–C_6_H_4_–CH_2_O–). ^13^C-NMR (chloroform-*d*_1_) ppm: 162.79 (C_4_), 131.94 (C_5_) (1,2,3-selenadiazole), 125.51, 129.23, 115.59, 159.12 (–C_6_H_4_)–(C_2_N_2_Se, 69.50 BrArCH_2_O, 135.84, 129.27, 135.5, 122.18 (Br–C_6_H_4_–CH_2_O–). FDMS *m*/*z*: 393.9 [M^+^, 100]. Analyzed percentage calculated for BrC_15_H_11_O N_2_Se: C 45.69, H 2.79; found C 45.59, H 2.73.

### 3.13. 4-[4-((4-Chlorobenzyl)oxy)-phenyl]-1,2,3-selenadiazole *(**6b**)*

Orange to reddish-brown solid, 63% yield, m.p. (°C): 160 (decomposed); IR (KBr) (cm^−1^): C–H at 3080, 2920, 2850; C=C at 1640, 1580; N=N at 1480, 1450. UV (chloroform) λ_max_: 257.5, 331 (nm). ^1^H-NMR (chloroform-*d*_1_) ppm: 9.28 (1H, s, 1,2,3-selenadiazole), 8.01 (2H, d, *J*_AB_ = 8.7 Hz, H_A,_ –C_6_H_4_)–(C_2_N_2_Se)), 7.10 (2H, d, J_AB_ = 8.7 Hz, H_B,_ –C_6_H_4_)–(C_2_N_2_Se)), 5.14 (2H, s, Cl–ArCH_2_O–), 7.39 (2H, d, *J*_AB_ = 8.8 Hz, H_A,_ Cl–C_6_H_4_–CH_2_O–), 7.42 (2H, d, *J*_AB_ = 8.8 Hz, H_B,_ Cl–C_6_H_4_–CH_2_O–). ^13^C-NMR (chloroform-*d*_1_) ppm: 162.63 (C_4_), 133.9 (C_5_) (1,2,3-selenadiazole), 128.77, 128.82, 115.42, 158.98 (–C_6_H_4_)–(C_2_N_2_Se), 69.31 ClArCH_2_O, 135.33, 129.1, 135.14, 125.33 (Cl–C_6_H_4_–CH_2_O–). FDMS *m*/*z*: 349.9 [M^+^, 100]. Analyzed percentage calculated for ClC_15_H_11_ON_2_Se: C 51.51, H 3.15, N 8.01; found C 51.41, H 3.10.

### 3.14. Antimicrobial Activity

The activity of the heterocyclic compounds **5a**, **5b**, **6a**, and **6b** was tested against certain human pathogenic microbes including Gram-positive *Staphylococcus aureus* (ATCC 29213), Gram-negative *Escherichia coli* (ATTC 35218), local resistant *Pseudomonas aeruginosa* and *Pseudomonas aeruginosa* (ATCC 27853), and *Candida albicans* (ATCC 10231) by the hole diffusion method, grown on nutrient agar.

The solutions of compounds **5a**, **5b**, **6a**, and **6b** were prepared by dissolving them in dimethyl sulfoxide (DMSO) as a solvent. A series of different concentrations were prepared to determine the lowest concentration that could affect the pathogen.

### 3.15. Genotoxicity Assessment

#### 3.15.1. Lethal Dose (LD_50_) Test for Compounds **5a** and **6a**

The animals used in the study were six-month-old naive Fisher male rats. The animals were reared in the animal care facility at Jordan University of Science and Technology (JUST), Irbid, Jordan, at 24 ± 1 °C. The study procedure was approved by the animal care and use committee at JUST. Rats were caged in polycarbonate cages with wire lids and ad libitum access to rodent chow and tap water. Rats were divided into seven groups/compound with six rats in each group. Compounds were dissolved in DMSO and administered to rats via intraperitoneal (i.p.) injection, using a 21-gauge needle. Control rats were injected with the vehicle only (DMSO). Animals were observed for 14 days by measuring general health, activity of animals, food intake, and appearance of sensitivity in the skin, increase or decrease in pupil diameter, and piloerection. The up-and-down procedure (UDP) was followed (OECD 425; OECD 1998).

#### 3.15.2. 8-Hydroxy-2′-deoxyguanosine (8-OHdG) Level in Urine

In this experiment, 40 male Fischer rats were divided into five groups, with each group consisting of eight rats**.** Compound **5a** was dissolved in DMSO to prepare doses of 0, 0.5, 5, 50, and 500 µg/kg of body weight (b.w.). Each group was treated with one of the doses via intraperitoneal injection, using a 21-gauge needle. The rats were injected at 12:00 p.m., and urine samples were collected from 8:00 a.m. to 3:00 p.m. The urine samples were stored at −20 °C immediately after collection. The same procedure was repeated for the other compound (**6a**)**.** Levels of 8-OHdG in urine samples were measured using a commercial ELISA kit (Stress Marq’s Company, Canada) that utilizes a 96-well plate coated with anti-mouse immunoglobulin G (IgG). Urine samples were diluted according to the kit’s instructions and then were added to the plate wells with the required reagent. The plates were incubated for 18 h at 4 °C, and then the absorbance was read at 405 nm using an ELx 800/universal microplate reader (bio-TEK instrument/USA). The 8-OHdG concentration was determined and normalized to urine creatinine level.

## 4. Conclusions

In this study, new 1,2,3-thiadiazole (**5a, 5b**) and 1,2,3- selenadiazole (**6a, 6b**) derivatives were prepared and screened in vitro for their antimicrobial activity against various pathogenic microbes. In addition, two compounds (**5a** and **6a**) were examined for their in vivo genotoxicity using an 8-hydroxy-2′-deoxyguanosine (8-OHdG) assay. Compounds **5a** and **5b** were found to be highly active against Gram-positive and Gram-negative bacteria. In addition, a significant inhibition of urinary 8-OHdG level (50.2%) was observed upon treatment of animals with 500 mg/kg b.w. of compound **6a** (*p* < 0.0001). The LD_50_ values for compounds **5a** and **6a** showed that these compounds are safe, since the LD_50_ was >5000 mg/kg b.w. Thus, the tested compounds might have the potential for use as antibiotics, since they showed strong antimicrobial activity and low toxicity.

## Figures and Tables

**Table 1:** Percentage yields of the synthesized compounds **3a**–**6a** and **3b**–**6b**.

Compound	Product	Yield (%)
**2a**	**3a**	98
**2b**	**3b**	97
**3a**	**4a**	85
**3b**	**4b**	82
**4a**	**5a**	98
**4b**	**5b**	97
**4a**	**6a**	60
**4b**	**6b**	63

**Table 2:** Sensitivity of human pathogenic microbes to the new synthetic heterocyclic compounds using the hole method.

Compound	Concentration (mg/mL)	*Staphylococcus aureus*	*Escherichia coli*	*Candida albicans*	*Pseudomonas aeruginosa*	*Pseudomonas aeruginosa* ATCC 27853
**5a**	10	+	−	+	−	−
**5b**	10	+	−	+	−	−
**6a**	10	−	−	−	−	−
**6b**	10	−	−	−	−	−

(−) Negative effect, (+) positive effect.

**Table 3:** Sensitivity of human pathogenic microbes at different concentrations of compounds **5a** and **5b.**

Compound	Concentration (µg/mL)	*Staphylococcus aureus* Inhibition Zone (mm)	*Candida albicans* Inhibition Zone (mm)
**5a**	1000	5	7
	500	3	5
	100	-	1
		
	5000	5	7
**5b**	2500	4	5
	1000	3	4
	500	1	3
	100	-	2

**Table 4:** The lethal dose (LD_50_) study of compound **5a.**

Cage	No. of Rats (*n*)	Dosage (µg/g) *	Clinical Signs after Injection	Clinical Signs after 14 Days	No. of Dead Rats
Control	6	0	Decrease in activity	Normal	0
C1	6	50	Decrease in activity	Normal	0
C2	6	100	Decrease in activity	Normal	0
C3	6	250	Decrease in activity	Normal	0
C4	6	500	Decrease in activity	Normal	0
C5	6	2000	Decrease in activity	Normal	0
C6	6	5000	Decrease in activity	Normal	0

(*) Dosage (µg) of compound **5a** per g of animal body weight (b.w.).

**Table 5:** The LD_50_ study of compound **6a**.

Cage	No. of Rats (*n*)	Dosage (µg/g) *	Clinical Signs After Injection	Clinical Signs after 14 Days	No. of Dead Rats
Control	6	0	Decrease in activity	Normal	0
S1	6	50	Decrease in activity	Normal	0
S2	6	100	Decrease in activity	Normal	0
S3	6	250	Decrease in activity	Normal	0
S4	6	500	Decrease in activity	Normal	0
S5	6	2000	Decrease in activity	Normal	0
S6	6	5000	Decrease in activity	Normal	0

(*) Dosage (µg) of compound (6a) per g of animal body weight.

## Supplementary Materials

The supplementary materials are available online.
