# Supplementary material for: Synthesis, Characterization, Antimicrobial Activity, and Genotoxicity Assessment of Two Heterocyclic Compounds Containing 1,2,3-Selena- or 1,2,3-Thiadiazole Rings
Source: Molecules. 2019 Nov 12;24(22):4082. doi: 10.3390/molecules24224082 (PMC6891806; doi:10.3390/molecules24224082)
Supplement: Supplementary file 1 [file molecules-24-04082-s001.pdf]

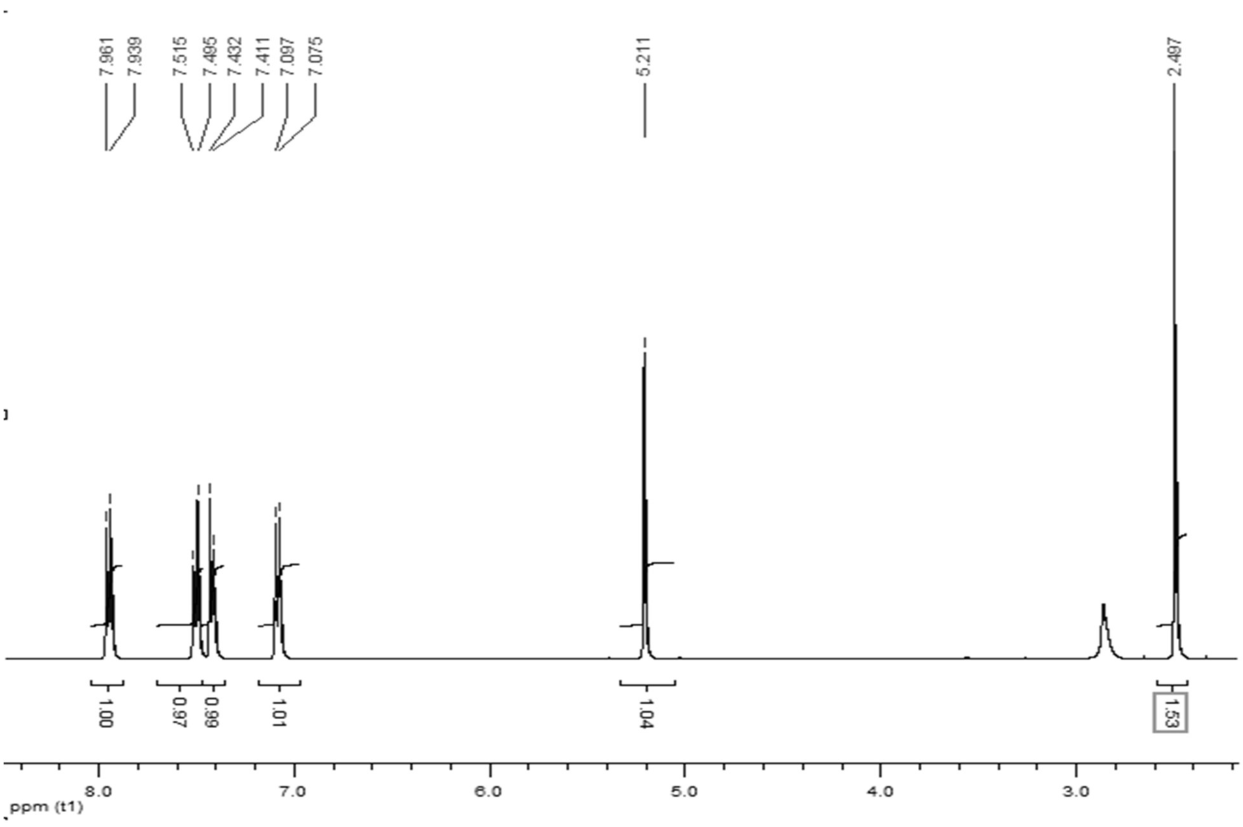

Supplementary figure 1: ^{1}H-NMR-spectrum 0f compound **3a** in acetone-d_6

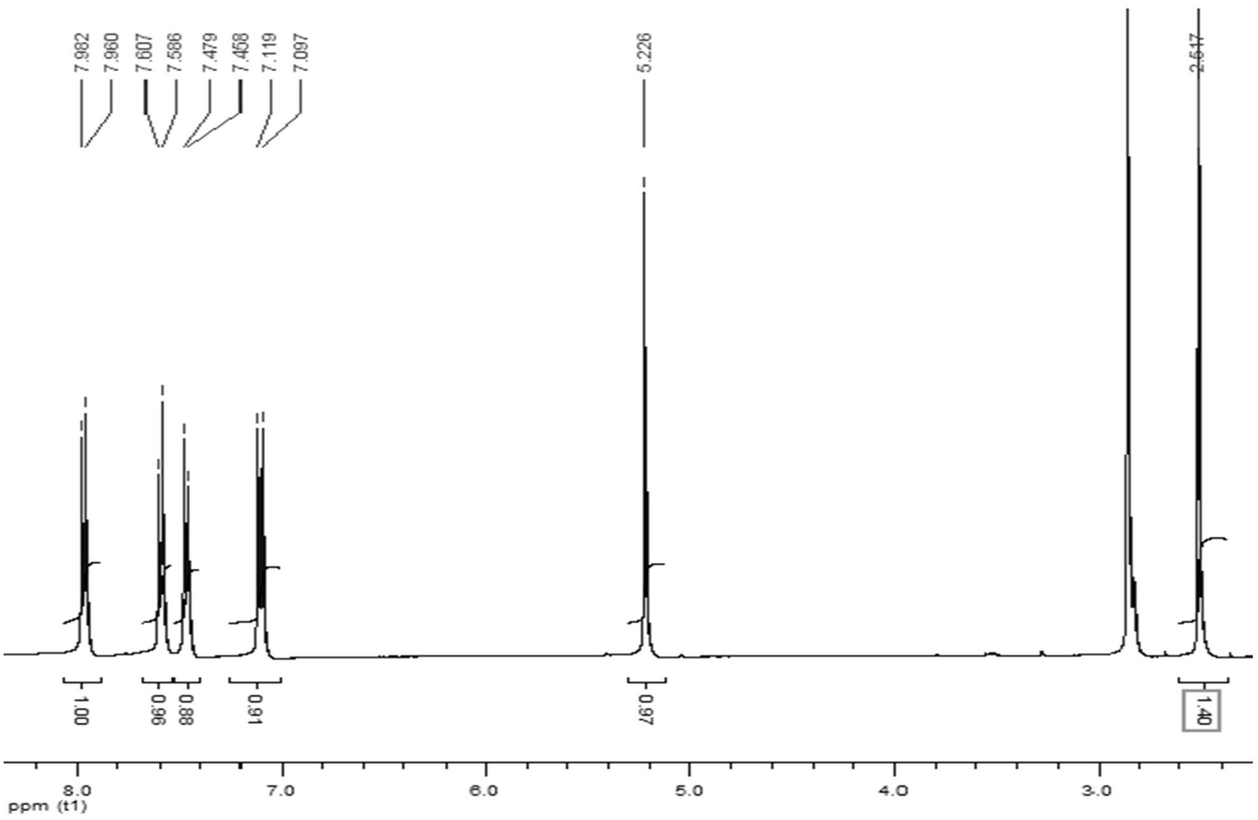

Supplementary figure 2: ^{1}H-NMR-spectrum 0f compound **3b** in acetone-d_6

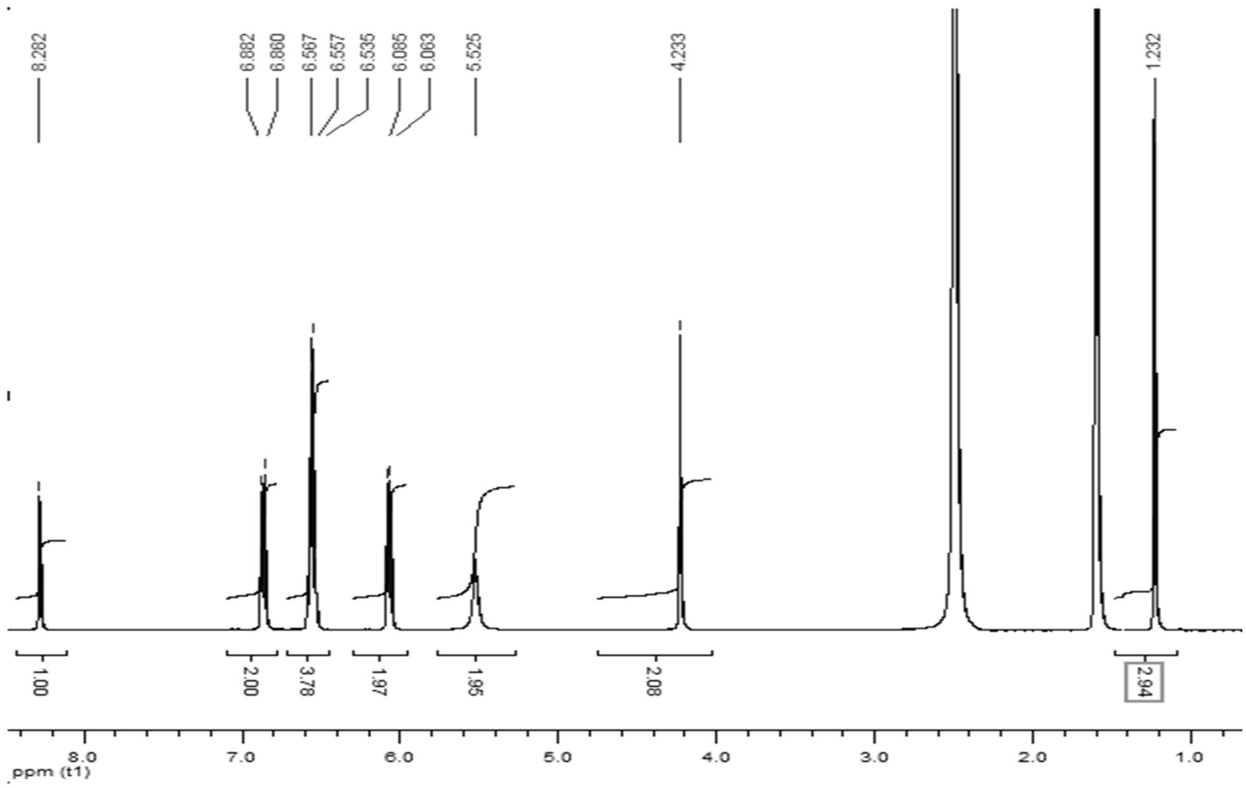

Supplementary figure 3:: ^{1}H-NMR-spectrum 0f compound **4a** in DMSO-d_6

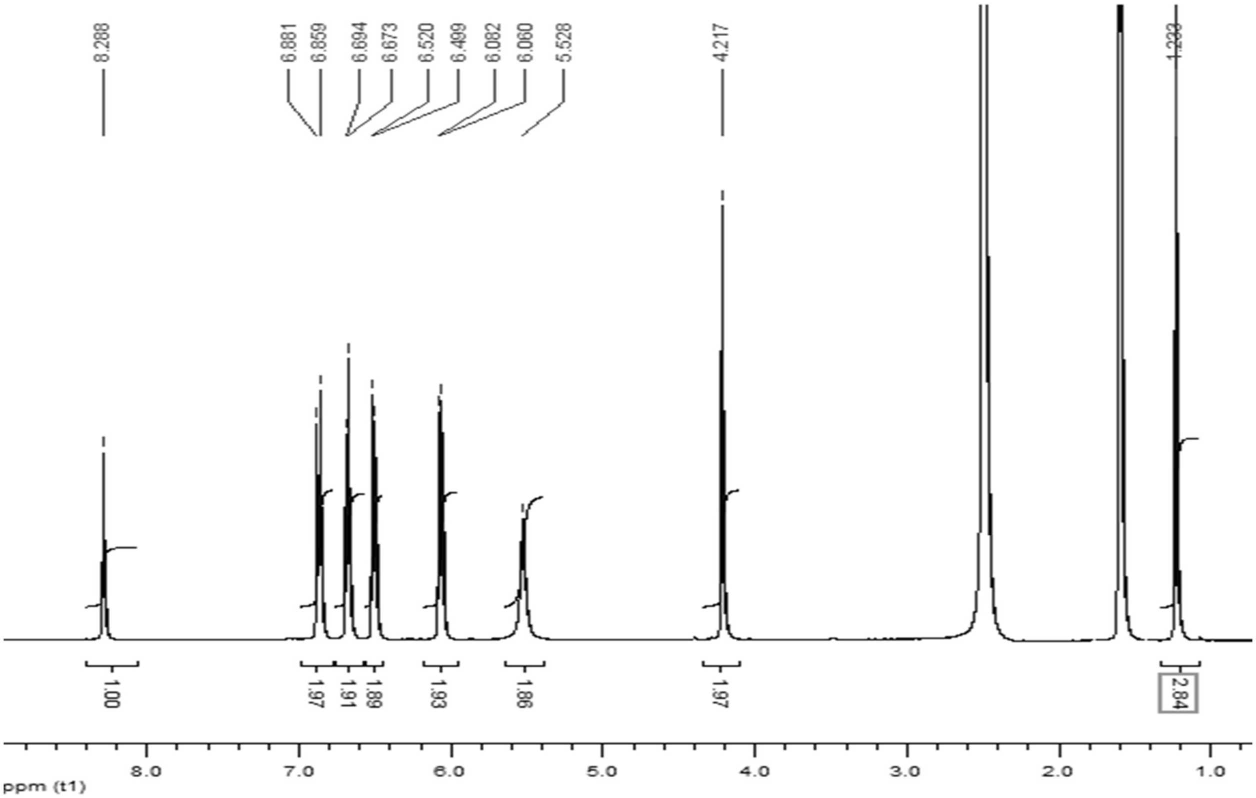

Supplementary figure 4:: ^{1}H-NMR-spectrum 0f compound **4b** in DMSO-d_6

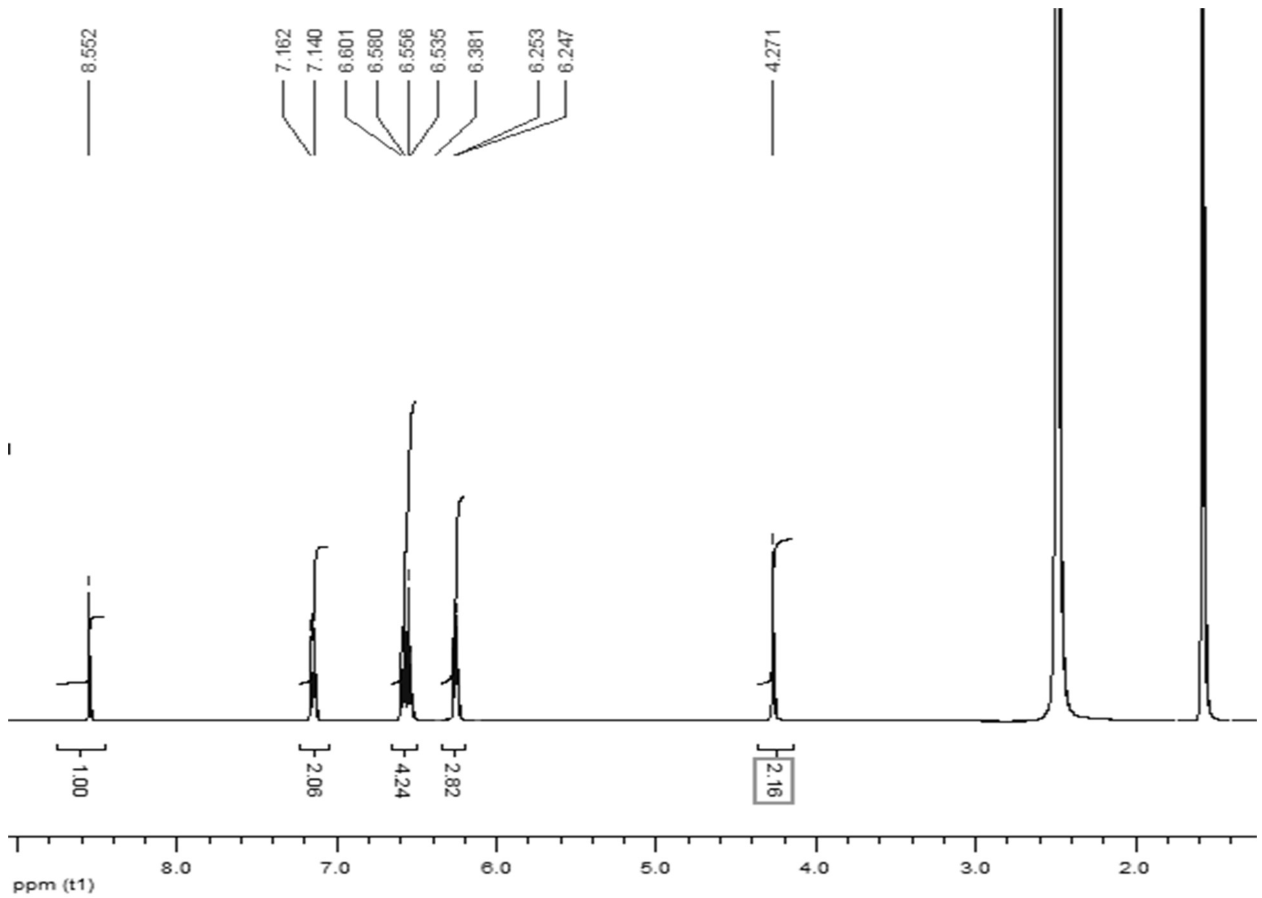

Supplementary figure 5: ^{1}H-NMR-spectrum 0f compound **5a** in DMSO-d_6

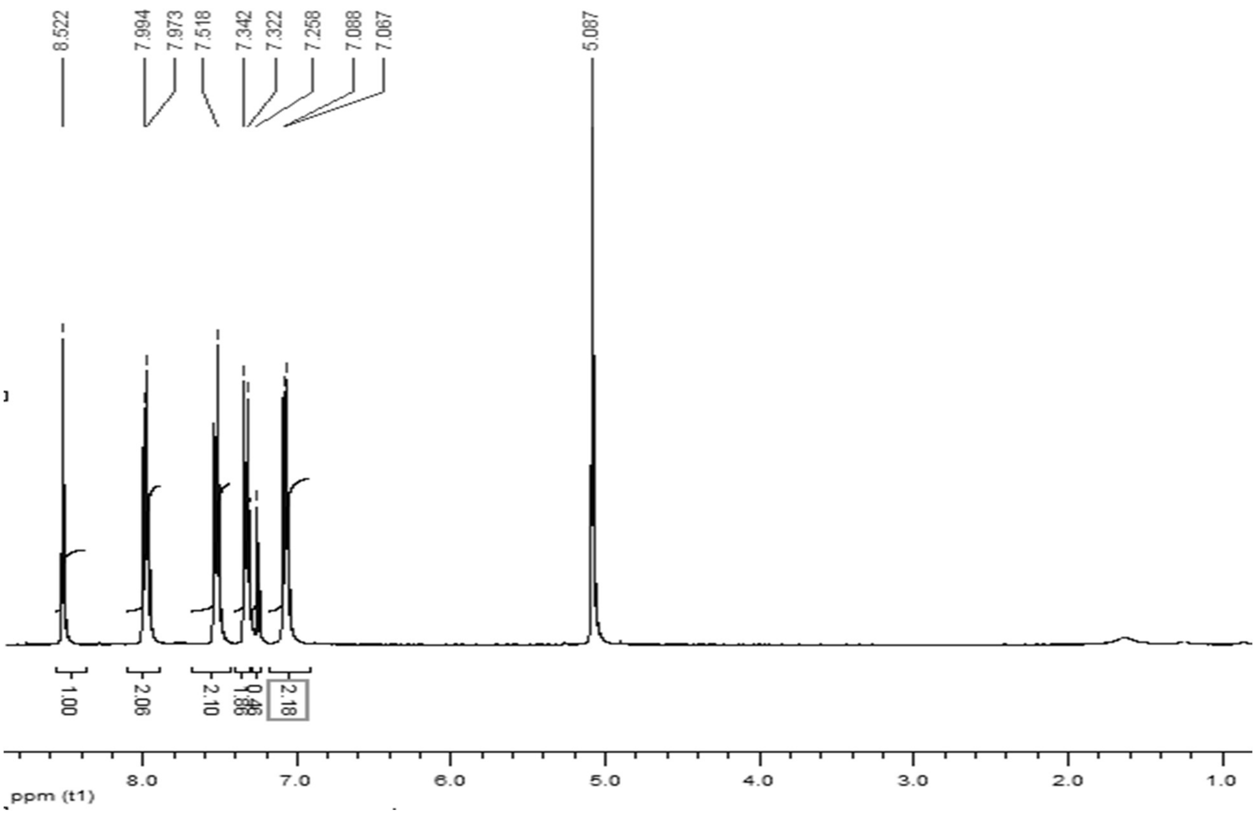

Supplementary figure 6: ^{1}H-NMR-spectrum 0f compound **5b** in $CDCl_3$

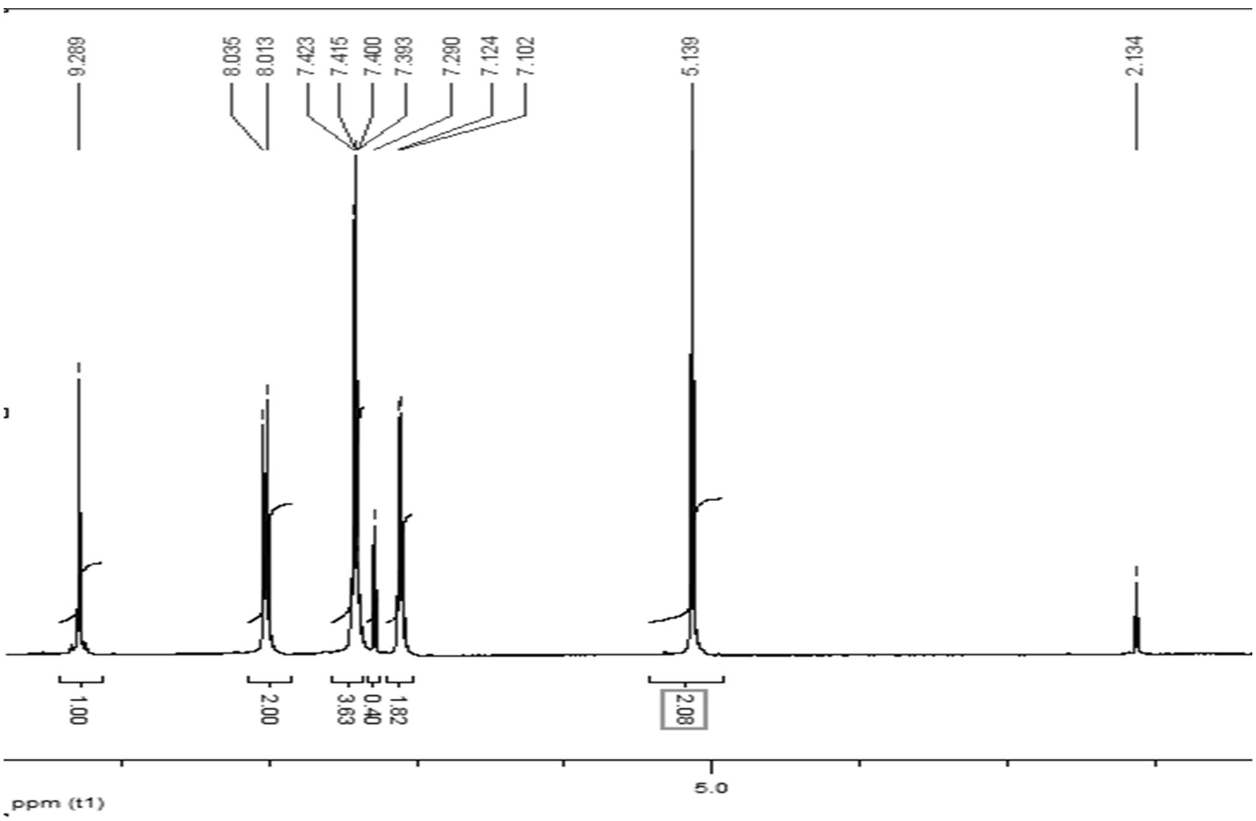

Supplementary figure 7: ^{1}H-NMR-spectrum 0f compound **6a** in $CDCl_3$

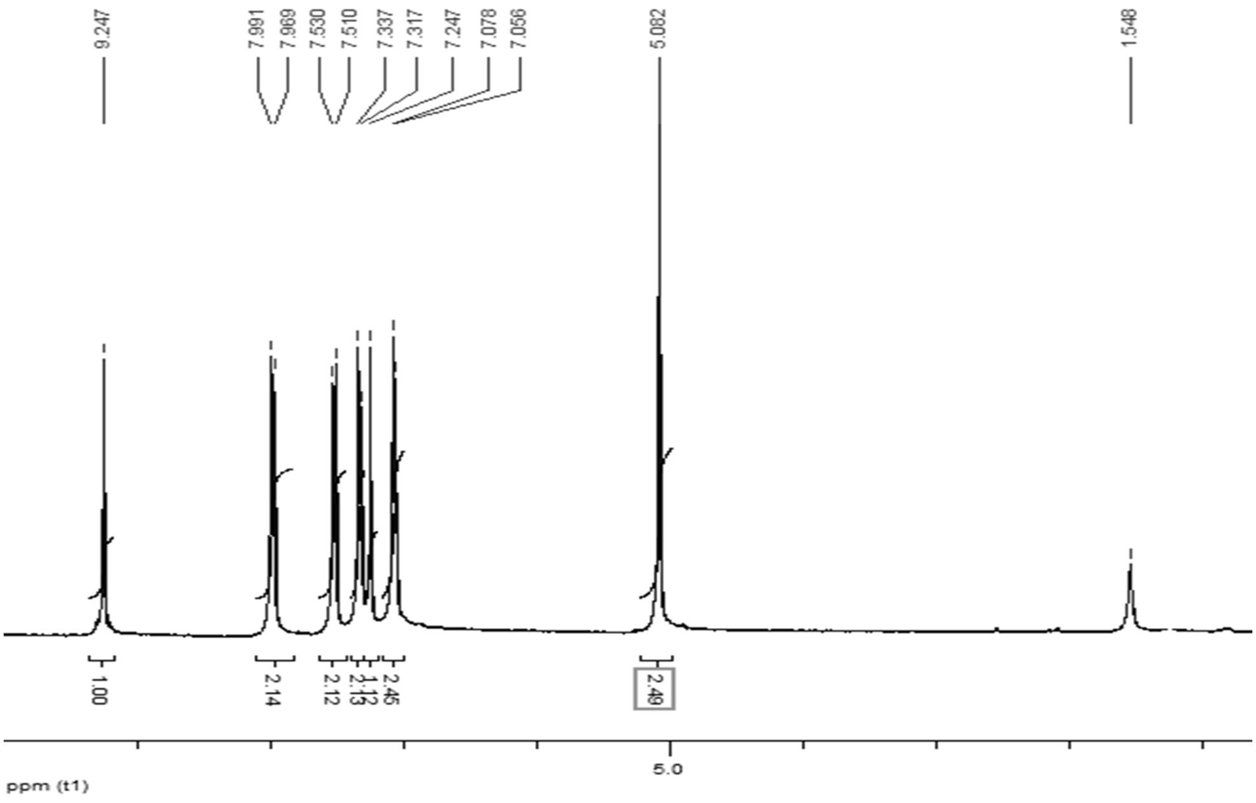

Supplementary figure 8: ^{1}H-NMR-spectrum 0f compound **6b** in $CDCl_3$

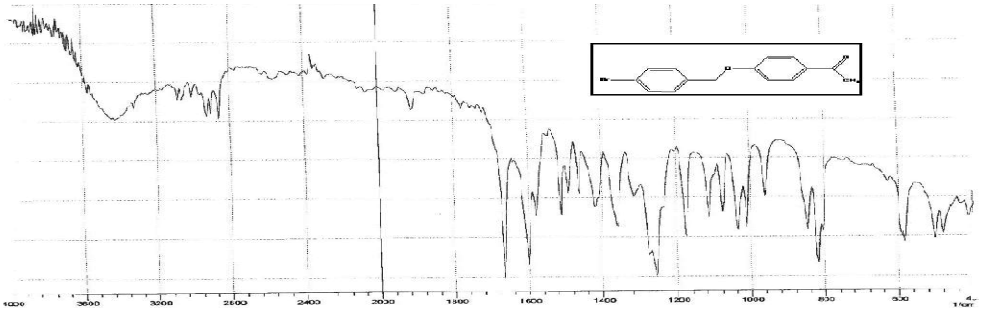

Figure 9: IR-spectrum 0f compound **3b** in KBr disc

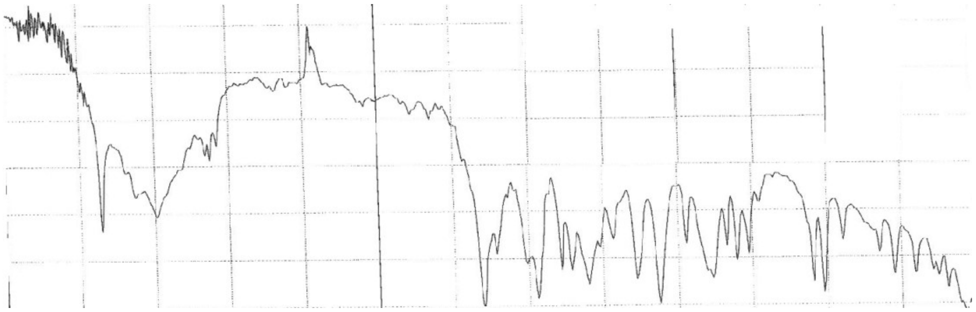

Supplementary figure 10: IR-spectrum 0f compound **4b** in KBr disc

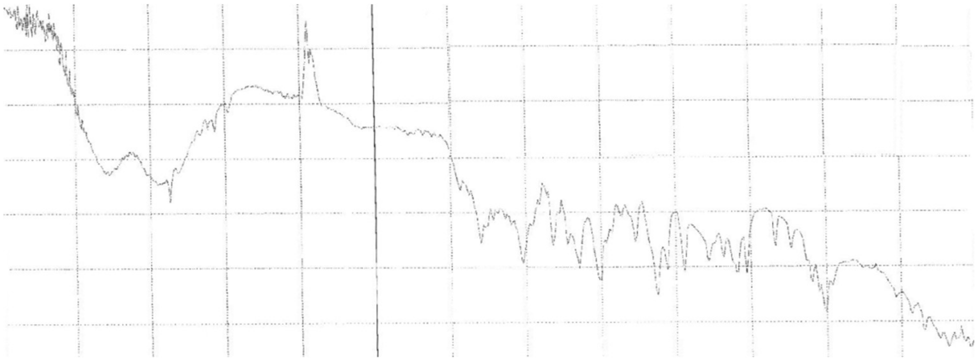

Supplementary figure 11: IR-spectrum 0f compound **5b** in KBr disc

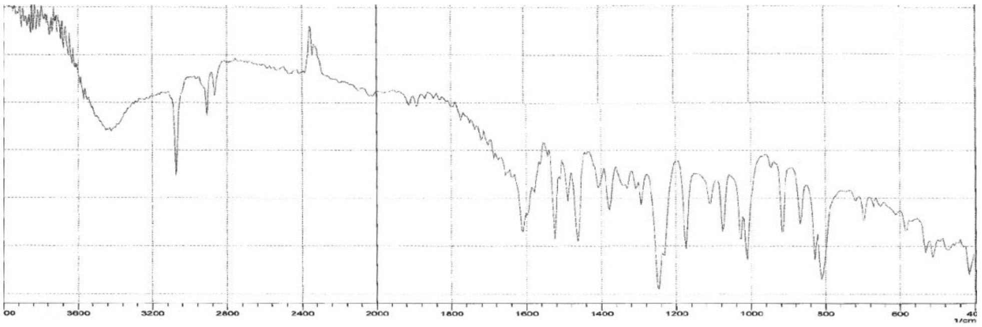

Supplementary figure 12: IR-spectrum 0f compound **6b** in KBr disc

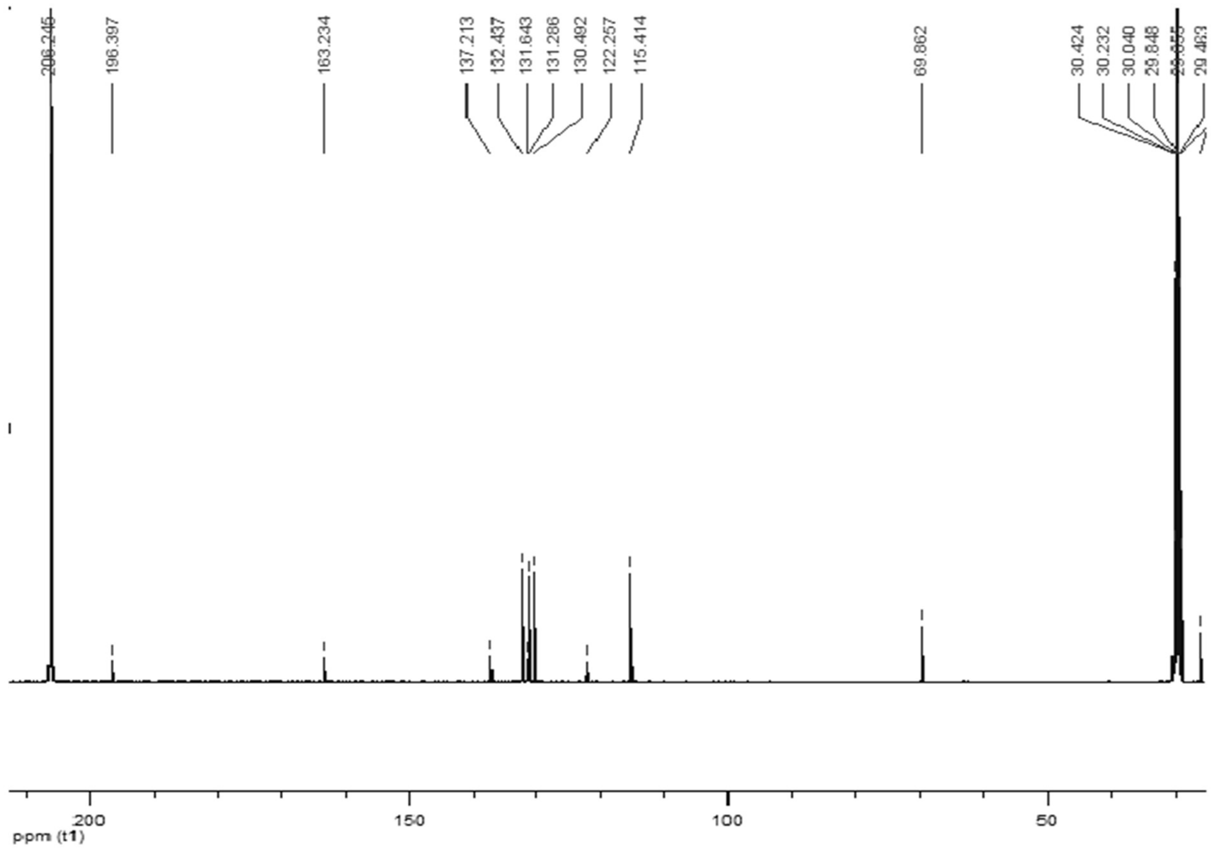

Supplementary figure 13: ^{13}C-NMR-spectrum 0f compound **3b** in acetone-d_6

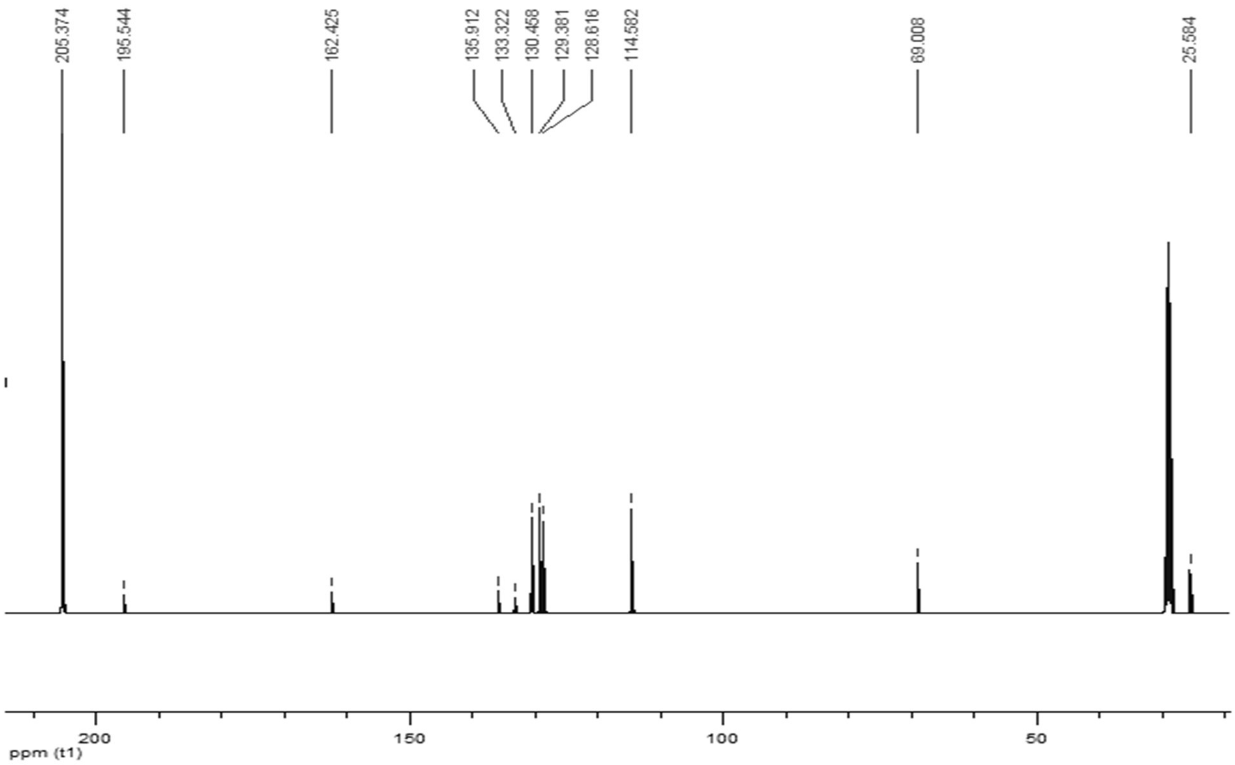

Supplementary figure 14: ^{13}C-NMR-spectrum 0f compound **3a** in acetone-d_6

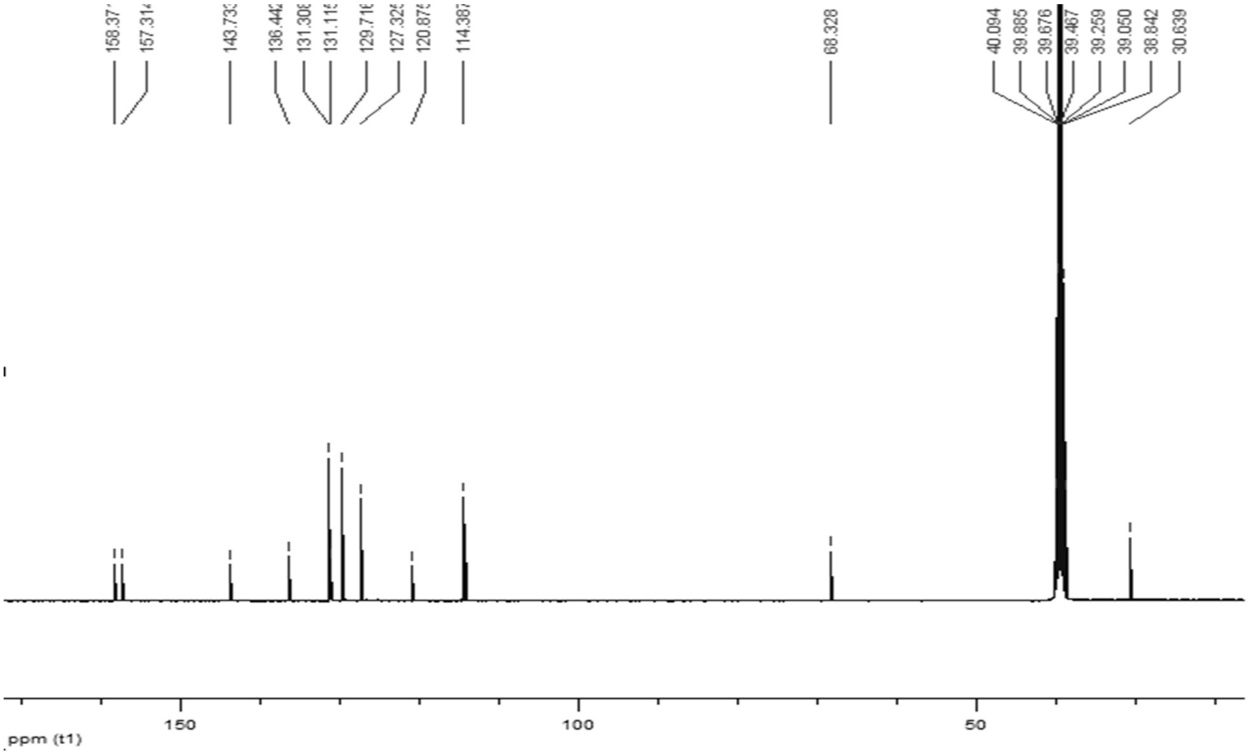

Supplementary figure 15: ^{13}C-NMR-spectrum 0f compound **4b** in DMSO-d_6

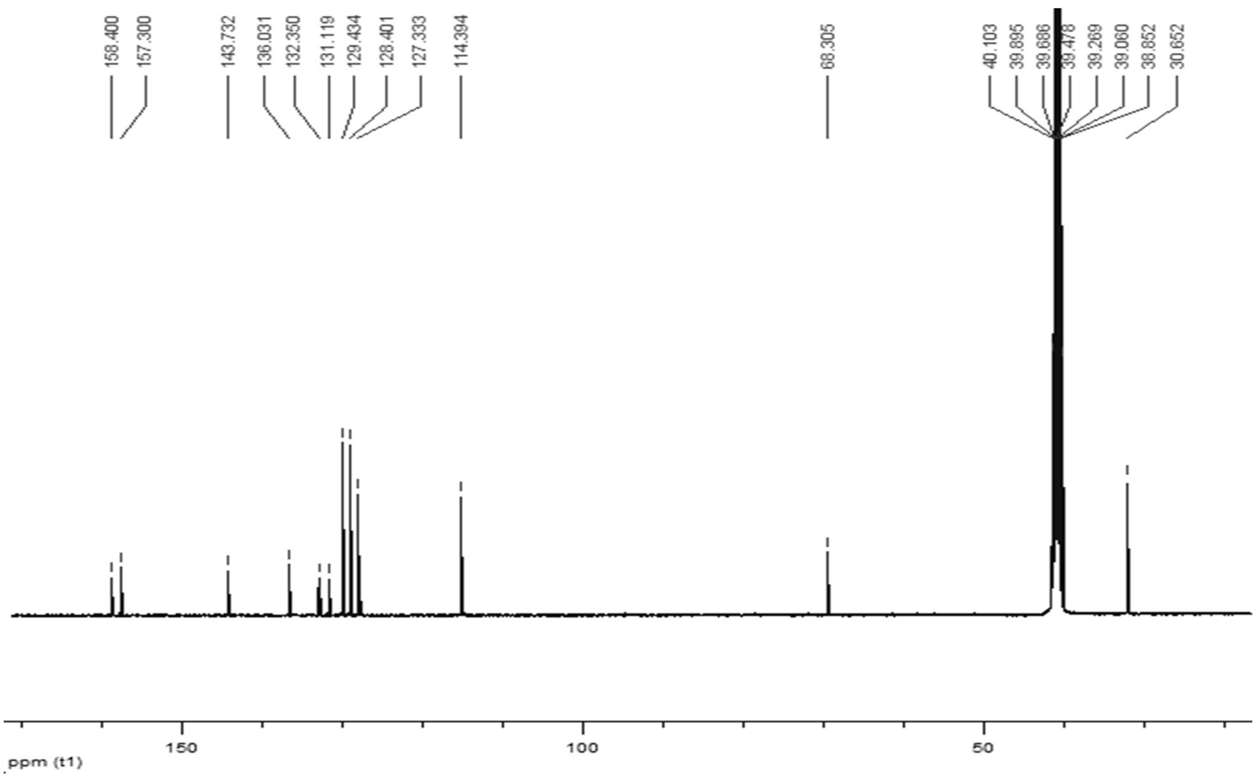

Supplementary figure 16: ^{13}C-NMR-spectrum 0f compound **4a** in DMSO-d_6

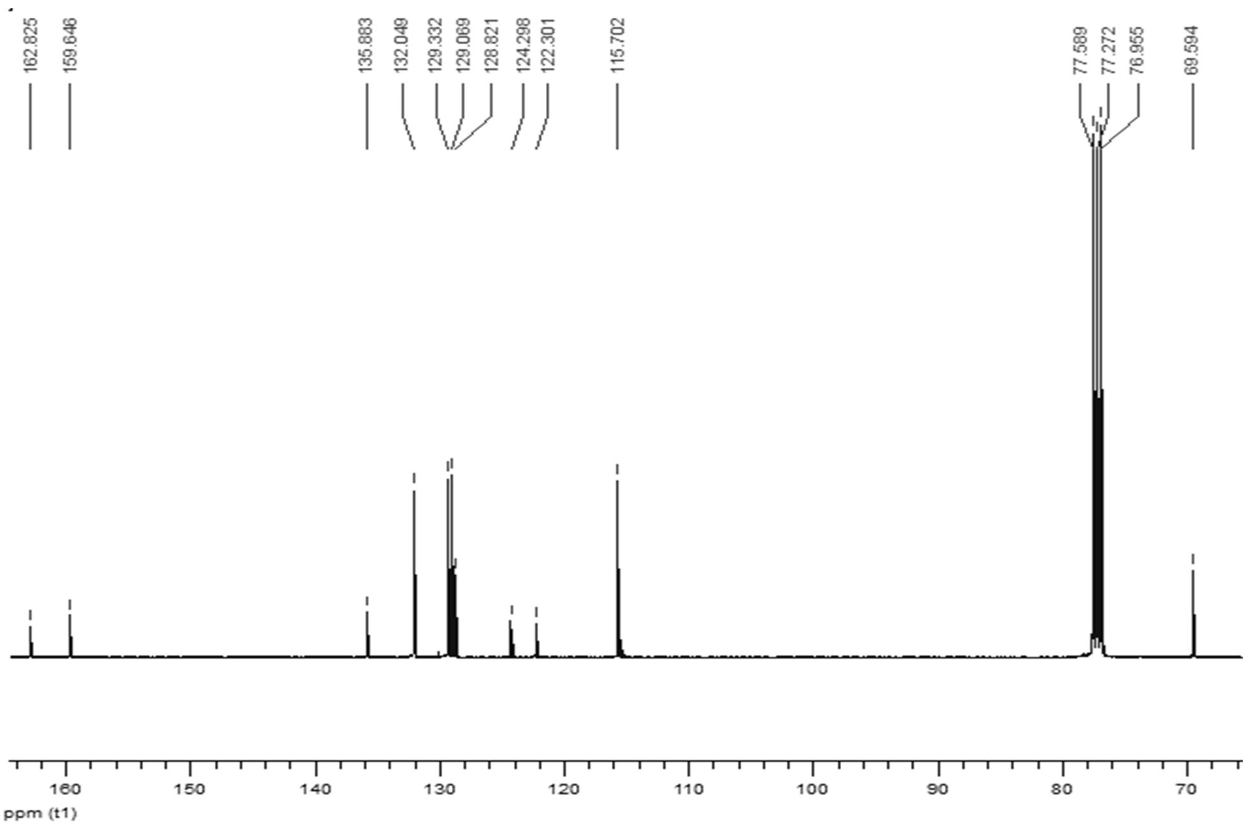

Supplementary figure 17: ^{13}C-NMR-spectrum 0f compound **5b** in $CDCl_3$

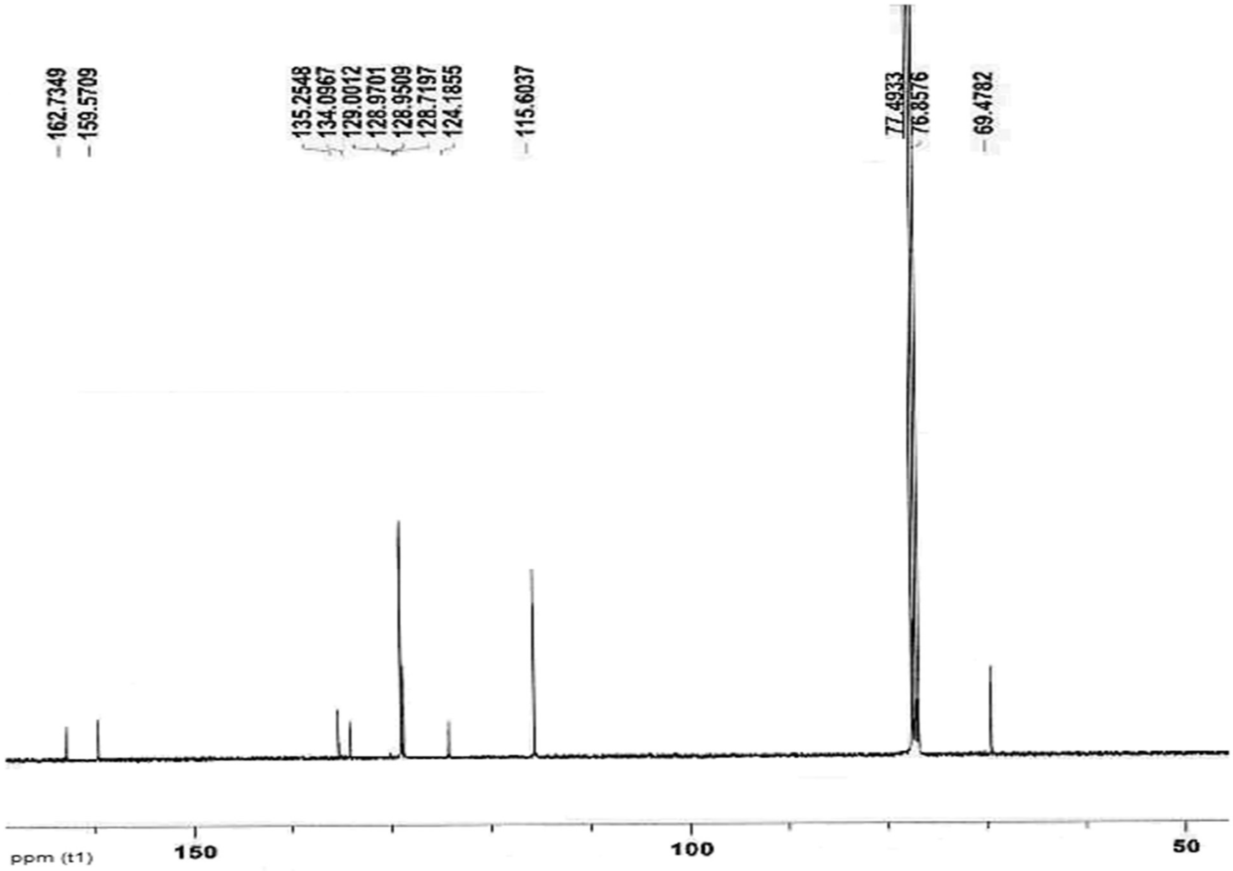

Supplementary figure 18: ^{13}C-NMR-spectrum 0f compound **5a** in $CDCl_3$

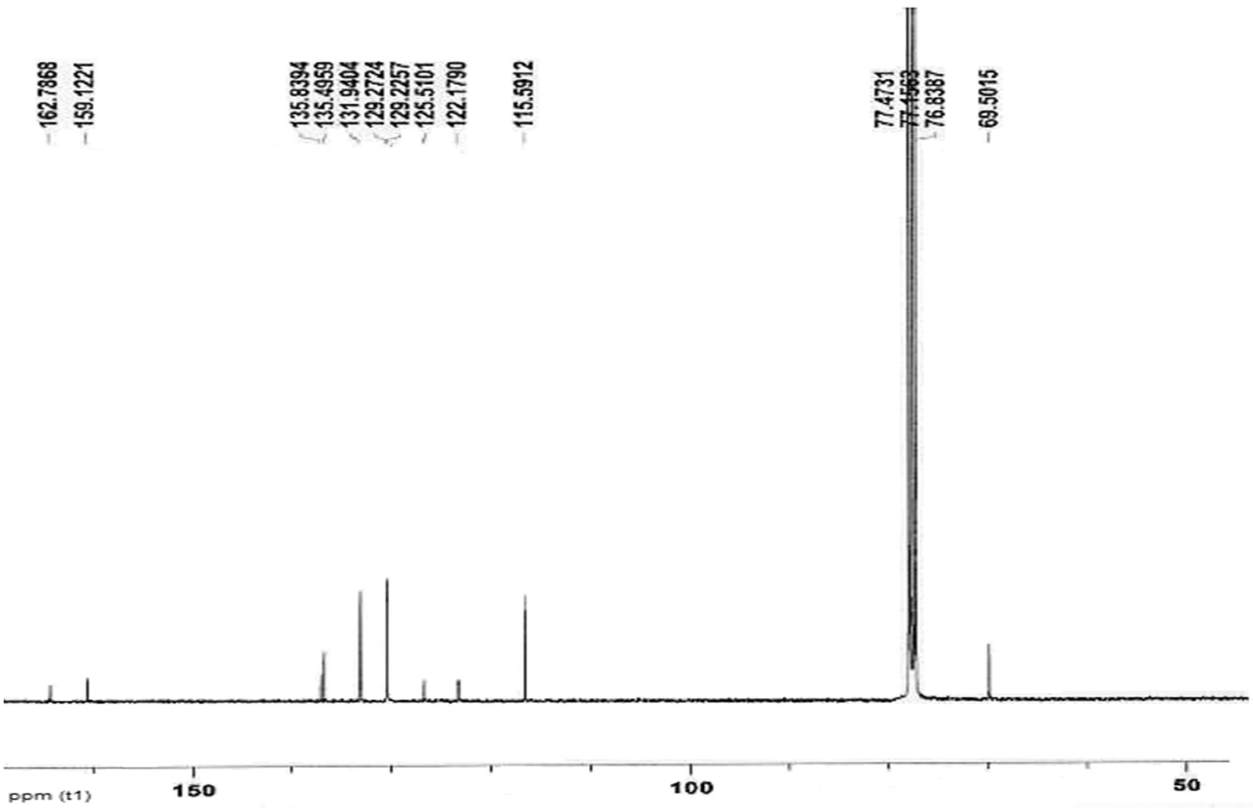

Supplementary figure 19: ^{13}C-NMR-spectrum 0f compound **6b** in $CDCl_3$

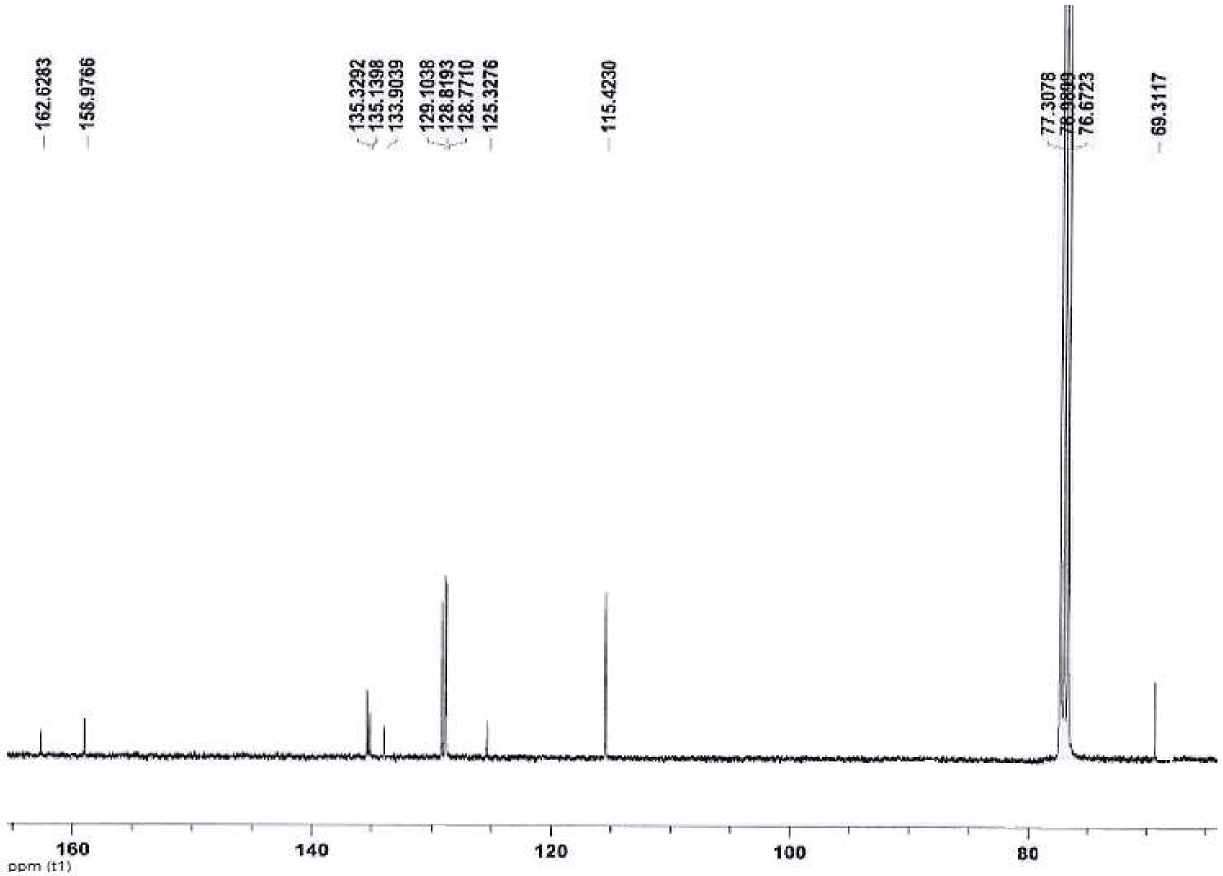

Supplementary figure 20: ^{13}C-NMR-spectrum 0f compound **6a** in $CDCl_3$
